# Key Components of Participatory Design Workshops for Digital Health Solutions: Nominal Group Technique and Feasibility Study

**DOI:** 10.1007/s41666-025-00199-4

**Published:** 2025-05-14

**Authors:** Kerstin Denecke, Octavio Rivera-Romero, Guido Giunti, Karin Van Holten, Elia Gabarron

**Affiliations:** 1 Department of Engineering and Computer Science, Institute Patient-Centered Digital Health, Bern University of Applied Sciences, Quellgasse 21, Biel, 2502 Switzerland; 2 Electronic Technology Department, Universidad de Sevilla, Seville, Spain; 3 Instituto de Ingeniería Informática (I3US), Universidad de Sevilla, Seville, Spain; 4 School of Medicine, Trinity College Dublin, Dublin, Ireland; 5 Faculty of Medicine, University of Oulu, Oulu, Finland; 6 School of Health Professions, Competence Centre Participatory Health Care, Bern University of Applied Sciences, Bern, Switzerland; 7 Department of Education, ICT and Learning, Østfold University College, Halden, Norway

**Keywords:** Participatory design, Workshop, Digital health, Framework, Co-design, Co-production

## Abstract

Participatory design (PD) is an essential method in the development of digital health solutions since it promises to increase acceptance, usability, and trust in the developed solution. Although careful planning and preparation is crucial for the success of PD workshops, a framework of key components to consider is still missing. The objective of this work is to develop such framework enriched with examples for aspects to be considered when planning and conducting PD workshops for designing and developing digital interventions in healthcare. We applied the nominal group technique with four participants with backgrounds in computer science, health informatics, psychology, and social anthropology to identify key components of PD workshops. The resulting framework was applied by an expert in PD to a case of a digital health solution for fatigue self-management for multiple sclerosis. The feasibility and applicability of the framework and its shortcomings were assessed. As a result, a framework consisting of five main categories and a total of 36 factors were assigned and defined in relation to the categories. The categories are participatory process, involved persons and their roles, workshop definition, setting, privacy and ethics, including regulations. The application of the framework to the test case demonstrated the feasibility and applicability of the suggested framework as well as the shortcomings of the analyzed PD process. This framework provides practical guidance while highlighting the complexity of PD workshops, encouraging their broad adoption, critical reflection, and continuous refinement. It has potential to improve the conduct of PD workshops and, in this way, potential to improve usability, acceptance, and usefulness of digital health solutions. In future work, the user perspective could be used to extend the framework.

**Supplementary Information:**

The online version contains supplementary material available at 10.1007/s41666-025-00199-4.

## Introduction

The use of digital tools such as apps, social media, websites, and chatbots is becoming increasingly common in healthcare, particularly in mental health, but also in other areas of healthcare dealing with a wide range of health challenges [1–3]. This trend is driven by the potential of digital health solutions to enhance patient care [4], improve health outcomes, and reduce healthcare costs [5]. However, there is a recognized risk that digital health solutions could actually exacerbate inequalities in the population, for example in terms of access, digital literacy, or language barriers [6]. Participatory design (PD) and co-design practices are critical to addressing these risks already in the development of digital health solutions [7, 8]. PD is a broad methodology that involves representatives of future user groups and experts to ensure that the solutions are easy to use, useful, and aligned with the needs and preferences of the target users. This approach is essential for gathering insights, generating ideas, and co-creating solutions that are contextually appropriate and user-centered [9]. It is based on the conviction that the specific expertise that users bring is a “valid form of knowledge” that adds valuable information to professional knowledge [10].

Workshops are not separate from PD, but rather one of its core mechanisms, providing a structured and collaborative environment where various stakeholders, including end-users, designers, and experts, can actively participate in the design process [11]. In PD, the goal is to involve all relevant parties in shaping the final product or intervention [8]. Participatory workshops offer a structured platform for gathering insights, generating ideas, and co-creating solutions [11]. They use a range of mechanisms to facilitate communication, foster creativity, and enable stakeholders to share their perspectives, preferences, and needs directly [11]. By engaging participants in workshops, designers can ensure that the resulting designs are more useful, user-centered, contextually appropriate, and aligned with the needs and preferences of the target users. Therefore, workshops are highly relevant in PD, as they enable meaningful collaboration and co-design efforts [11].

When it comes to digital health, PD becomes even more critical to ensure that these interventions are effective, culturally sensitive, and accessible [8, 12]. By embedding the unique perspectives and needs of end-users, researchers and stakeholders from an early stage of the design process, PD promotes solutions that resonate deeply with users’ experiences and needs [8, 12]. This increases both reach, uptake, and engagement, potentially leading to greater intervention effectiveness [13, 14]. A review on digital approaches for the participatory development of health-related interventions presented by Doerwald et al. [15] only focuses on digitally conducted workshops. Our work goes beyond and considers any PD workshop aimed at designing or developing digital health solutions, conducted in person or digital. We are not reviewing approaches, but identifying key components for their successful conduct and developing a guideline as a practical tool for considering these components in PD workshops.

Despite the growing use of PD in healthcare, there is a lack of comprehensive guidelines for planning and delivering PD workshops. Existing studies often report on specific methodologies and logistical aspects but do not provide a standardized framework for the design and delivery of PD workshops. For example, research on digital mental health interventions have proposed various frameworks or guidelines for integrating evidence-based approaches for co-designing mental health interventions. Examples of these frameworks include the one proposed by the “A Young and Well Cooperative Research Centre” for codesigning online youth mental health interventions [8]. This proposed framework includes six components: identify (e.g., identify the problem from the perspective of young people and the evidence base); define (e.g., identify the beneficiaries and define the problem space and objectives with them); position (e.g., understand how the intervention needs to be positioned and framed in order to have an impact for young people and relevance to young people); concept (e.g., identify, generate and evaluate potential concepts that represent what the intervention needs to be and do to engage young people and deliver appropriate mental health and wellbeing outcomes), create (e.g., evolve, build and refine an intervention that is useful and usable by young people and safe and effective from mental health and wellbeing perspective); and use (e.g., deliver, use, evaluate and iterate the intervention based on how young people experience and use the intervention and its impact) [8]. A review listed a series of best practices and recommendations for undertaking PD of digital mental health interventions specifically with indigenous young people [16]. Suggested best practices are structured along four pillars: governance (i.e., address a need identified by the community); engagement (i.e., engage young people in an iterative process design, development and review); partnerships (i.e., create an environment of mutual learning to ensure capacity strengthening); and knowledge translation (i.e., demonstrate beneficial outcomes for individuals and community) [16].

The IDEAS (integrate, design, assess, share) framework guides a researcher through the full life cycle of an mHealth project [17]. Farao et al. combined the Information Systems Research framework with design thinking principles as a framework for mHealth application design [18]. Another study referred to the use of Design and Evaluation of Digital Health Interventions (DEDHI) framework [19] for planning workshops aimed at adapting a group-based intervention for trauma [20].

These existing frameworks are relevant for PD of digital health solutions; however, they do not provide specific guidance on how to conduct PD workshops in the area of digital health. To our knowledge, there is a lack of structured guidelines for effectively planning and conducting PD workshops in this sensitive and complex area. Several studies that have used participatory workshops for designing digital health solutions report on the methods used, focusing on the activities that they planned, the stakeholder engagement, or logistical aspects without offering a comprehensive framework or standardized guidelines [18, 21–25]. Since some relevant aspects of the PD process are not reported in these frameworks, researchers seeking to use PD in digital health solutions face significant challenges in replicating and adapting these approaches. In addition, effective planning and facilitation of a PD workshop are critical to ensure that participation meaningfully informs the design process rather than becoming a superficial exercise. In this context, the framework presented here provides a structured method for designing participatory activities within the implementation process.

A framework for conducting PD workshops in digital health is essential for several reasons. While PD methodology provides the overarching approach, workshops are the main medium for stakeholder involvement and co-creation. In addition to the challenges that any PD workshop can present, working with vulnerable populations presents unique challenges. Ethical and privacy considerations must be paramount, and participatory workshops must be conducted with compassion, fostering a safe and supportive environment. This study aims to develop a framework enriched with practical examples of the key components of PD workshops that lead to successful digital health solutions. In doing so, it aims to provide a guide that supports the implementation of PD workshops where participation is truly at the forefront and where, as a result, PD improves the effectiveness and user acceptance of digital health solutions, ultimately contributing to better health outcomes and user satisfaction.

## Methods

### Framework Development Methodology

**Fig. 1:**
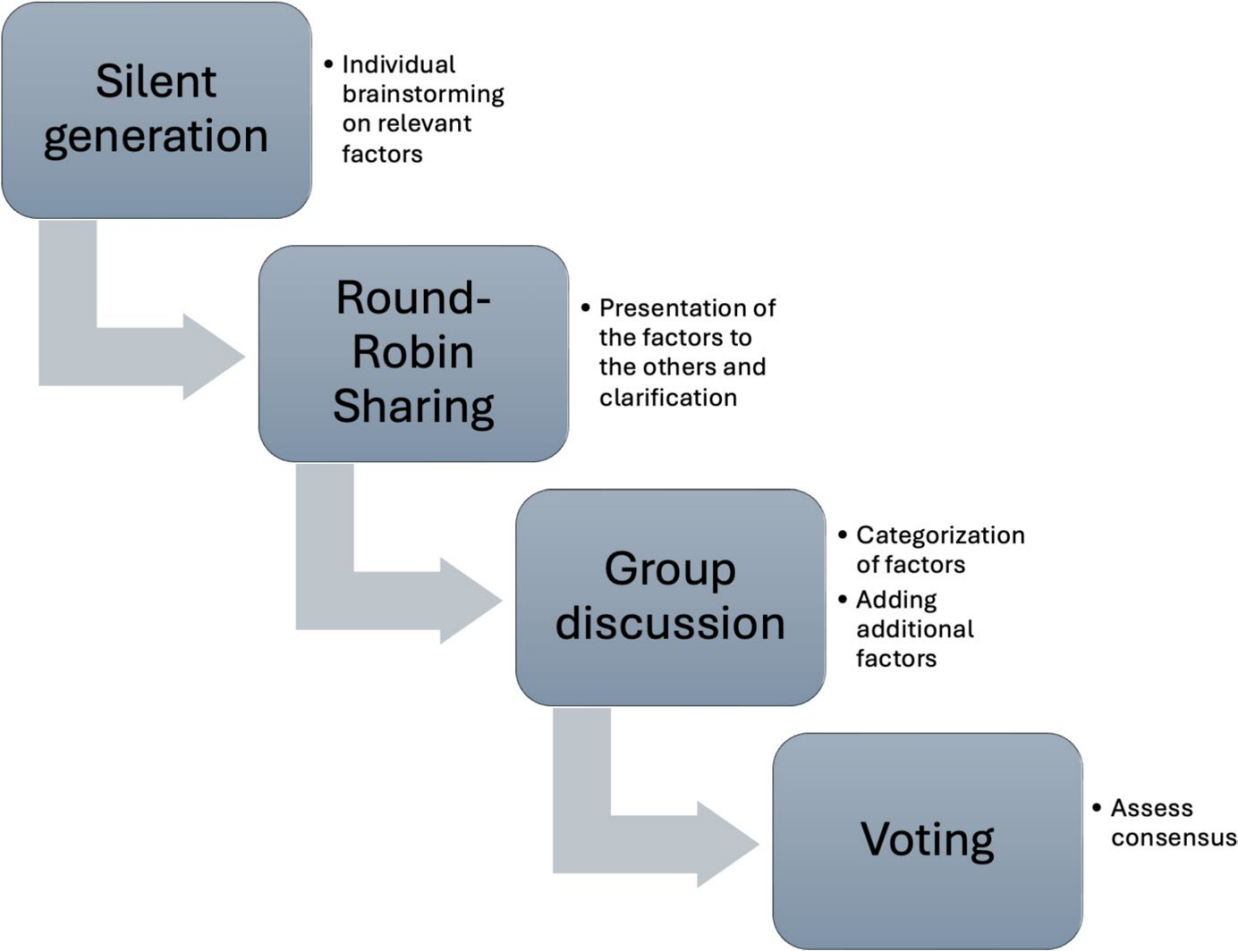
The nominal group technique was applied to collect factors relevant for PD workshops and categorize the factors

To achieve our goal, we conducted a two-steps approach: First, we identified key components of PD workshops using the nominal group technique [26, 27] (see Fig. 1). Second, we defined and specified the identified key components by group discussion.

The nominal group technique is a structured approach that fosters consensus among participants, commonly employed for priority setting in healthcare and research [26]. For our in-person meeting, we involved one social anthropologist, one psychologist, and two computer scientists. All persons are experienced in designing digital health solutions and in using participatory methods for this purpose. In a first round, these four participants applied silent generation [27] and collected individually key factors of PD workshops based on their experience in designing, conducting, or participating in PD workshops (time frame 10 min). The group conducted the round-robin sharing to collect and display all factors. Then, they elaborated and clarified the collected factors (sharing phase), with new components added as needed during this process. Specifically, each participant explained their factors, while the other participants listened carefully. All factors were collected at a Miro board [28]. In the following discussions, all factors were categorized, new factors were included, and redundant factors were removed (group discussion). The individual factors and categories were specified in detail with the aim of reaching a common understanding in order to finally formulate definitions. Therefore, the four researchers went through all the factors again together, agreed on the labels, and checked the assignments to categories. A voting was conducted to assess consensus. All voted factors were included in the final version.

### Framework Test Case

The feasibility and applicability of the framework were assessed by an expert in PD who was not involved in the process of defining the framework. This expert is a medical doctor, an expert in digital health, and has strong experience in PD. First, this researcher reviewed the framework and provided feedback on understandability of the categories and key components defined, missing components and relevance. Then, this researcher was asked to reflect on how the PD process followed in the test case might have changed based on the knowledge generated and lessons learned in each participatory workshop developed in the project if this framework had been employed.

The framework was explored by applying it to the case of a digital health solution for fatigue self-management for multiple sclerosis (MS) called more stamina [29–31]. More stamina is a gamified fatigue self-management solution developed through an iterative user-centered design that followed an evidence-driven approach [30, 32] guided by patient and public involvement (PPI) process [33]. Qualitative studies with healthcare professionals and people with MS were used to identify needs for digital health [34]; systematic reviews explored the mHealth landscape [35, 36]; early health technology assessment informed development [37]; and the value for healthcare professionals was explored [38]. Finally, its feasibility and usability were tested through real world use with people with MS [39].

While more stamina is primarily a tool for managing physical symptoms of MS, it addresses fatigue, a common symptom in both MS and many mental health conditions, showing the framework’s potential to be applied to other health contexts. Its impact in reducing fatigue, stress, and improving quality of life all contribute to better mental health outcomes for its users. The design and development process of more stamina is presented chronologically to provide context, followed by an analysis of how the different elements of the framework might have modified its design and development.

## Results

### Key Components of PD Workshops

The silent generation and round-robin phases led to ten categories which are environmental factors, planning, setting, data collection and analysis, recruitment and selection, participatory process, regulations, participant, workshop conductors, and responsible person. In the clarification phase, we did some changes to these initial categories to remove redundancies and ensure clarity and consistency of the categories:

- The category “planning” was changed to “workshop definition.”
- “Environmental factors” was removed as category and added as factor to “setting.”
- “Data collection and analysis” was moved to the category “workshop definition.”

All factors were considered relevant to be included in the framework in the voting activity. The suggested final framework for PD consists of five categories that are listed and defined in Table 1. In the following subsections, we are describing the factors per category.

**Table 1:** The main categories of the framework for PD consist of five categories

Participatory process	This category comprises factors that might impact the participation of participants, including participation barriers, plurality of opinions, and kind of expertise (diversity)	• Plurality of opinion • Plurality of expertise • Justification of the approach • Ownership • Joint rules • Joint terminology • Joint goals • Confidence in participants • Privacy protection • Participation barriers • Social fields of power • Impact of status and roles • Expectation management
Involved persons and their roles	This category includes the different persons involved in a PD workshop including their roles, characteristics, motivation, and literacy	• Participant characteristics • Participant motivation • Literacy (health literacy, digital literacy) • Empowerment of participant
Workshop definition	This category includes all organizational aspects related to the PD workshop. For example, the purpose and goals to be achieved, structure of the workshop, budgeting, or selection of suitable methods	• Purpose • Goals • Participatory planning • Role definition and tasks • Specific needs • Inclusion/exclusion criteria • Validation of inclusion/exclusion criteria • Workshop structure • Selection of suitable methods • Selection and preparation of material • Specification of the setting • Agenda • Budgeting, first check • Budgeting, second check
Setting	This category describes the environment of the PD workshop, including the appropriate material, expectation management, and environmental factors	• Availability of appropriate material • Environmental factors
Privacy and ethics including regulations	This category includes aspects related to ethics, ethics approval, and reflection on possible ethical issues as well as regulatory compliance of the workshop	• Ethical considerations • Ethics approval • Regulation compliance

Each category comprises several factors

### Participatory Process

The category “participatory process” consists of 13 factors to be addressed: plurality of opinions, plurality of expertise, justification of the approach, ownership, joint rules, joint terminology, confidence in participants, privacy protection, participation barriers, social fields of power, impact of status and roles, expectation management, and joint goals.

*Plurality of opinions* refers to the inclusion and respectful consideration of a wide range of perspectives, ideas, and viewpoints from all participants within a PD workshop. This means that all opinions are given equal weight. To realize this, an environment has to be created where diverse opinions are welcomed and valued and facilitate open discussions to ensure all voices are heard.

*Plurality of expertise* refers to the inclusion and integration of diverse types of knowledge, skills, and perspectives from a wide range of participants. This concept is crucial for fostering a holistic and comprehensive approach to the PD process. It also points to the goal of diversity, i.e., we might include users with the same chronic condition but with different socioeconomic status or other diversity criteria that might be relevant. When plurality of expertise (i.e., diversity) is not achieved, this must be reflected and justified.

*Justification of the approach* comprises an explanation why applying a PD process is required for achieving the given aim or answering a given research question. It also includes to ensure that participants understand the benefits and goals of the approach.

*Ownership* is a factor that should be achieved in the participants of PD workshops as it is a sense of responsibility, engagement, and interest that participants feel toward the process and outcomes of the workshop. This is essential to achieve relevant outcomes and engagement of the participants. Participants should be encouraged to take ownership of the process and outcomes and be involved in decision-making.

*Joint rules* are a set of agreed-upon guidelines and principles that govern the behavior, interactions, and processes within the PD workshop. These rules are, at best, collaboratively developed and, at minimum, accepted by all participants to ensure a respectful, productive, and inclusive environment. All participants should follow *joint goals*. These are shared objectives and desired outcomes that are collectively established and agreed upon by all participants. These goals guide the collaborative efforts and ensure that everyone is working toward common purposes.

A *joint terminology* is essential for ensuring understanding. It comprises a shared set of terms and definitions that are understood and used by all participants. This common language helps ensure clear and effective communication, reduces misunderstandings, and promotes a cohesive understanding of key concepts and processes.

The factor *confidence in participants* refers to the trust and belief in the abilities, insights, and contributions of all individuals involved. This could be fostered by providing support and encouragement to boost participant’s confidence.

*Privacy* *protection* consists of measures and practices implemented to safeguard the personal and sensitive information of all participants. All sensitive information should be handled with care and confidentiality.

Inclusion of all participants is essential. This is reflected by the factor *participation barriers*. These include any obstacles or challenges that prevent or hinder individuals from fully engaging in the workshop activities. These barriers can affect the inclusivity, effectiveness, and overall success of the workshop.

Additionally, *social fields of power*, i.e., social relations and their impact on the dynamics between participants, need to be considered collectively, i.e., by all participants.

*Impact of status and roles* refers to factors that are related to role and status models and how they influence the participants’ interaction in the workshop (e.g., hierarchies in hospital staff). The impact of status and roles should be acknowledged, and opportunities should be created for all participants to contribute in a meaningful manner.

Finally, expectations of all participants have to be managed. *Expectation management* is the continuous process of clearly communicating and aligning the goals, roles, responsibilities, and anticipated outcomes among all participants. It helps ensure that everyone involved has a shared understanding of what to expect, reducing the risk of misunderstandings and fostering a positive and productive workshop experience.

### Involved Persons and Their Roles

The category “involved persons and their roles” takes into account four relevant factors: participant characteristics, participant motivation, literacy, and empowerment of participants. *Participant characteristics* comprise the characteristics of the participants to make them eligible to join the workshop. Besides of recognizing and valuing the diversity of participant’s backgrounds, experiences and perspectives, activities and discussions should be tailored to accommodate different preferences. Inclusivity should be ensured by considering factors such as age, gender, cultural background, and abilities.

*Participant motivation* refers to the personal motivations of the participants to join the workshop. These should be understood and addressed to keep participants engaged. A positive and supportive environment that fosters intrinsic motivation should be created.

*Literacy* describes specific skills the participants must possess in order to be eligible for participating. This can include not only health literacy but also digital literacy. It might be necessary to provide material and training to enhance participant’s literacy skills. Clear and accessible language, avoiding jargon and complex terminology would also help to address different literacy levels.

The factor *empowerment of participants* refers to the psychosocial process of fostering relevance and self-efficacy, enabling individuals to gain the skills and confidence needed to actively and successfully engage in the workshop. Participants should be encouraged to take an active role in the PD process. A safe space should be created where participants feel confident to express their ideas and opinions.

### Workshop Definition

Defining the PD workshop (category “workshop definition”) requires consideration of 14 factors: purpose, goals, participatory planning, role definition and tasks, specific needs, inclusion/exclusion criteria, validation of inclusion/exclusion criteria, budgeting and first check on budget, second check on budget, workshop structure, agenda, selection of suitable methods, selection and preparation of material, and specification of the setting.

The *purpose* of conducting a PD workshop stands at the beginning of the workshop definition phase and outlines the intended outcomes and the reasons for conducting the PD workshop. Clearly define the purpose of the workshop to ensure all participants understand its significance. Its purpose should be communicated effectively to align everyone’s expectations and efforts.

For the PD workshop, *goals* to be achieved have to be specified. Specifically, goals should be specific, measurable, achievable, relevant, and time-bound (SMART). Additionally, the goals should be aligned with the overall purpose and objectives of the PD workshop.

*Participatory planning* refers to the collaborative process of planning and decision-making of the workshop process. This fosters also the sense of ownership and commitment.

*Role definition and tasks*: Roles and tasks per role have to be defined. This refers to the process of clearly identifying and assigning specific responsibilities and activities to each participant involved in the workshop. This process ensures that everyone understands their part in the workshop and contributes effectively to achieving the workshop’s objectives.

Participants may have *specific needs*, i.e., peculiarities of participants that might impact the workshop planning and conduct.

The factor *inclusion and exclusion criteria* refers to criteria that characterize the participants as eligible to join the workshop. They have to be defined and later in the process of participant recruitment be validated.

*Validation of inclusion/exclusion criteria* refer to the process of checking the participant’s characteristics to ensure they are eligible to join the workshop.

*Budgeting and first check on budget* and *second check on budget* are important to ensure that the necessary financial resources are available: Conducting a PD workshop requires a budget. We distinguish a first and a second check on the budget. The first check on the budget refers to all financial considerations needed to ensure the workshop can be conducted. The second check on the budget refers to the process of approving the available budget is suited for conducting the workshop as planned.

The *workshop structure* refers to the planned sequence of activities and sessions that guide the workshop from start to finish. This structure ensures that the workshop runs smoothly, covers all necessary topics, and achieves its objectives effectively. The workshop structure impacts on the agenda.

The *agenda* is a detailed schedule and outline of activities, sessions, and discussions planned for the duration of the PD workshop. It serves as a roadmap for both facilitators and participants.

*Selection of suitable methods:* Depending on the workshop structure, participants, purpose, and goal, suitable methods have to be selected. This factor refers to the process of assembling methods to be applied within the workshop to achieve the defined goals.

*Selection and preparation of materials:* Required material has to be selected and prepared carefully (e.g., prototypes, paper, pen). Well-prepared material ensures smooth execution of the workshop.

*Specification of the setting* refers to the detailed planning and description of the physical or virtual environment in which the workshop will take place. This includes considerations of location, layout, tools, and atmosphere to create an optimal space for collaboration and creativity.

### Setting

Within the category “setting,” we aggregate two factors: availability of appropriate material and environmental factors.

*Availability of appropriate material*: Within a PD workshop, various materials such as prototypes and documentation are required to run the workshop. These materials must be carefully assembled and available at the start of the workshop.

*Environmental factors* are various physical, social, and contextual elements that can influence the effectiveness, comfort, and outcomes of the workshop, e.g., accessibility, barrier-free, options for resting. A comfortable and conducive environment has to be chosen for the workshop, considering factors like lighting, seating, and acoustics.

### Privacy and Ethics Including Regulations

Within the category of privacy and ethics, we distinguish three factors: reflection on ethics, regulation compliance, and ethics approval.

*Ethical considerations *need to be made, i.e., ethical principles and issues that may arise during the PD workshop need to be discussed and considered. The health, well-being, safety, and rights of participants have to be ensured throughout the process. This also includes ensuring transparency and honesty in all communications and actions. Further, the potential impacts of the workshop on participants and the broader community should be considered.

*Regulation compliance:* Beyond considering ethical principles, PD workshops have to be compliant with relevant regulations, laws, standards, and guidelines. This ensures that the workshop operates within legal and ethical boundaries, protecting the rights and well-being of participants and stakeholders.

*Ethics approval* is required to conduct PD workshops. It is the process of obtaining formal permission from an ethics review board or committee to conduct the workshop, ensuring that the planned activities adhere to ethical standards and principles.

### Framework Test Case

To illustrate the practical application of the proposed framework, we reflect on the design and development process of the more stamina project. Below, we describe how the different factors of our framework could have been considered to support the various stages of the more stamina design and development process.

## Conceptualization

The process to identify fatigue as a primary debilitating symptom for people with MS that would benefit from the design of a digital health solution was conducted by an exploration of the scientific literature as well as through focus groups and interviews with healthcare professionals and people with MS. Half of the participants were female, with median age of 43.5 years, reflecting the typical age distribution of persons living with MS. The cohort was relatively educated, with most having completed higher education qualifications and was still predominantly working population, though some experienced challenges related to employment because of MS.

The level of impact on this population’s quality of life and activity levels demanded for some sort of self-management tool. Systematic reviews of available digital health solutions highlighted the lack of condition-specific mHealth apps for MS, particularly ones tailored to energy management with gamified engagement. During the conceptualization phase, low-fidelity mock-ups and paper prototypes were created.

- *Plurality of opinion and expertise:* The framework emphasizes the importance of including a wide range of perspectives. At this point in the cases’ journey, only persons with MS and healthcare professionals who worked with them were considered relevant stakeholders. Members of the people with MS’ family and social circle were not involved in the process. By systematically ensuring the inclusion of these other stakeholders, the project could have benefited from even broader insights.
- *Justification of the approach:* Also at this point, PD was not used in full, but rather as a philosophy guiding the exploration. The main goal had been to understand the needs of the target population and not constructing a solution for it. Explicitly articulating why PD was essential could have strengthened participants’ understanding of their role, increasing engagement and ownership.

### Prototype Design

Co-creation sessions were conducted with representatives of different stakeholders to produce user requirements and features. These representatives were software engineers, interaction designers, persons with MS, nurses, neurologists, physiotherapists, and family doctors. Deriving insights obtained from interviews, focus groups, and co-design sessions, various concepts were devised and refined. People with MS and healthcare professionals were involved in brainstorming and prototyping to strive for better alignment with their needs. Once a definitive concept was established, an interactive prototype was designed by an experience interaction designer who had been present in all sessions. The interactive prototype was then iteratively tested for usability using Nielsen’s usability testing and Wizard of Oz approaches.

### Purpose and Goals

- Each co-creation session was kept independent, with the output of one session not being used as input for another. This was done at this stage in order to obtain the largest possible number of design concepts that attempted to address fatigue management. As such, there were a significant number of concepts that had to be discarded because they were not feasible or realistic (e.g., a robot that would follow them around and do their chores for them).
- Defining clear purposes and goals for each workshop stage could have provided participants with a stronger understanding of the objectives.
- *Confidence in participants:* Entering the creative mindset and proposing designs was challenging for some of participants as the approach was too novel for them. For example, some healthcare professionals struggled with ideation because they wanted to be told what the “right design” was, while software engineers tended to focus too much on the specifics of a feature without conceptualizing a broader view. Encouraging trust in participants’ abilities would reinforce their confidence, potentially eliciting deeper insights.
- *Expectation management:* During the different sessions, it was challenging for some of the participants to navigate the level of concreteness that their prototypes should have. Clearly communicating expectations could have aligned goals and reduced potential frustrations.
- *Social fields of power and impact of status and roles:* Sessions that blended a mix of backgrounds created situations that the facilitators had to resolve and navigate. Healthcare backgrounds were seen as more authoritative even if the participant had never treated a person with MS before. The different roles in the healthcare team were often not understood by non-healthcare participants, leading to assumptions and biases. Being mindful of hierarchical dynamics between healthcare professionals and users might have helped in balancing contributions, ensuring all voices were equally heard.

### Development

Development followed a frugal innovation and agile approach, prioritizing requirements based on cost, time, and resource availability to produce a functional product. A PPI team was formed to have ready access to users’ input, and patients were hired as user representatives who were part of the more stamina team. The PPI team was involved as co-design partners and strategy board members. A series of issues emerged from the use of PPI as team members in a complex project and setting such as unclear task division and reimbursement schemes. At times, PPI members were not sure what was expected of them and resorted to a more passive stance, simply responding to questions when asked. In terms of reimbursement schemes, it became problematic clarifying what sort of tasks were to be compensated, and which were part of the general team spirit. Once such issue occurred when a PPI member decided to promote the project through social media and then presented an invoice for services rendered. A working digital health product was developed using a combination of ad-hoc solutions and third-party components.

- *Ownership and joint goals:* Establishing joint goals and fostering a sense of ownership might have further motivated the PPI team, leading to more meaningful contributions.
- *Participant characteristics and motivation:* It would have been useful to understand the specific reason why these people with MS joined our PPI team to further personalize a set of tasks for them. People with MS interested in the design aspects could have joined the SCRUM sprint goal setting, while those more interested in advocacy could have joined the communications team. By thoroughly profiling participants’ characteristics and motivations, the project could have tailored activities to better suit their needs and interests. Clearly defining roles and tasks could have optimized collaboration and efficiency.
- *Environmental factors:* During activities that involved people with MS, venue accessibility, comfort, and amenities were considered to improve participant well-being and engagement.

### Testing

A pilot study was conducted in Finland to assess the feasibility, acceptability, and usability of more stamina where people with MS utilized the solution for 60 days in real world conditions. Think-aloud protocols and structured interviews were conducted to assess user feedback, as well as tracking usage patterns through the solution itself. Over the 2-month period, as participants engaged with the solution and integrated its use into their daily lives, new uses emerged. People with MS showed more stamina statistics and graphs to their family members as a means of increasing their mutual understanding of fatigue and sharing the burden. The need for a “companion” version of the solution emerged as users wished to share the management of their condition with their loved ones. While the overall experience of participants was positive, the methodology used for the pilot imposed additional tasks on people with MS, which was reflected on in the post-interviews.

- *Plurality of opinion and expertise:* As was mentioned in the conceptualization stage, if family members of people with MS had been involved earlier in the process, the need for a companion version may have been identified earlier. Streams of parallel design processes with a shared vision could have strengthened the final version of the digital solution.
- *Participatory planning:* Involving the PPI team in the planning process of the pilot study might have increased engagement and ensured activities met the expectations not only of PPI members but also of the persons with MS who were subjects of the study.

## Discussion

### Principal Results

This paper introduces a comprehensive framework that outlines essential components for designing a PD workshop aimed at creating and developing digital health solutions. The framework encompasses five categories and 36 key components, addressing the participatory process, the roles and contributions of involved individuals, workshop planning and structure, contextual setting, and considerations for privacy and ethics. A checklist is provided as practical tool for considering these key components in the PD workshop planning and conduct (see Appendix 1).

### Application of the Framework to the Digital Solution More Stamina

The evaluation of the framework in the test case demonstrated its feasibility and practical applicability. In this context, the lead researcher identified several challenges that arose during the design process of more stamina that could have been anticipated and addressed if the proposed framework had been implemented. Specifically, these challenges relate to aspects that are crucial for fostering a common understanding between the research or development team, users and domain experts. Although the more stamina team aimed to include the perspectives of users and experts in their development process, their early involvement in the workshop planning was lacking. This omission limited the opportunities for participants to articulate their needs, thereby undermining the participatory nature of the process.

### Comparison with Prior Work

A multitude of studies have reported the importance of involving stakeholders in the design process of digital health solutions and interventions [33, 40–42]. The benefits of using these PD methods have led many studies focusing on the design of these types of solutions or interventions to use these types of participatory methods. Although there are several design frameworks that are frequently used in the design of digital health solutions or interventions [17, 18, 25, 43–46], most of these establish high-level aspects, such as the stages of the design process or concrete methods of participation in specific domains. The proposed framework goes a step further by identifying key components for the design of a specific participatory activity in the design of digital health solutions or interventions. These considerations are in line with recommendations from other studies [42, 47–49]. In this regard, Vial et al. recommend explaining the rationale behind adopting a human-centered design approach, which is aligning with the “justification of the approach” component in our framework [42, 49]. Voorheis et al. addressed participant characteristics, joint terminology, plurality of expertise, and ethics in their work [48]. Lastly, Yrttiaho et al. shared insights from involving patients in digital health research, focusing on participant characteristics, plurality of expertise, joint terminology, role definitions, and participation barriers [47]. The framework presented here integrates these factors and puts them in a systematic order to give guidance to those planning and leading a PD workshop.

### Implications for Research and Practice

The proposed framework supports researchers in planning and conducting PD workshops aimed at developing digital health solutions, ensuring that all relevant aspects are thoroughly addressed.

The practical implications of the PD framework for developing digital health solutions center on creating an inclusive, ethical, and structured environment that actively supports and values participant engagement. Practitioners are encouraged to foster a culture of openness and mutual respect, embracing diverse opinions and expertise and ensuring that all voices are heard and valued. Clear communication of the workshop’s purpose, goals and participatory approach helps to build trust and alignment, while jointly developed terminology, goals, and rules promote shared understanding and commitment. By involving participants in planning and decision making, the process fosters ownership and empowerment, which increases both engagement and motivation. Attention to participants’ characteristics—such as literacy levels, backgrounds, motivations, and specific needs—ensures accessibility and relevance, while a thoughtful approach to managing power dynamics and role expectations promotes equitable participation. Practical aspects such as defining roles and inclusion criteria, choosing appropriate methods and materials, structuring the workshop, and maintaining a conducive environment all contribute to smooth and effective implementation. Ethical and legal considerations, including privacy, ethics approval, and regulatory compliance, need to be prioritized throughout. Ultimately, this framework emphasizes the importance of adaptability, preparation, and participant-centered design to achieve meaningful and impactful outcomes in digital health innovation. These recommendations are provided as checklist in the Appendix 1.

By adopting the framework and practical guidance, PD workshops can facilitate meaningful participation, enabling the participatory process to produce practical, well-accepted digital solutions. To be effective, the workshop design process itself must be participatory from the outset, actively involving users and experts. However, this approach can be challenging, particularly in research projects, as it requires additional resources and time to align potentially conflicting individual goals. In addition, the findings of this study can inform the creation of reporting guidelines for studies using PD methods in the development of digital health solutions or interventions, promoting transparency and consistency in the application of these methods.

### Limitations

This study has some limitations that are reported below. First, the number of experts involved in the definition of the framework is small and has a limited expertise representation, which may have limited the diversity of perspectives and generalizability of the key components. The inclusion of further experts with different experiences in the PD process in digital health and, in particular, of user experts could lead to the identification of other factors that have not been included in the current version of the framework yet. Furthermore, the framework was developed without direct involvement of the target population (i.e., users of digital health solutions) and therefore potentially not capturing real-world needs and preferences of the intended users. On the other hand, a validation has been performed based on the experience of a single expert and only one project. It is clear that this cannot ensure the understandability and completeness of the definitions and the framework. More extensive validation is needed to ensure completeness. As the relevance of PD of digital health solutions is increasingly recognized, we believe it is important to provide guidance as the proposed framework does, and to expand and improve it continuously by incorporating real-world experiences from its application.

## Conclusions

PD workshops are a powerful approach to creating digital health solutions that are both needs-driven and user-centered. By drawing on the collective expertise of professionals experienced in PD workshops, this framework aims to provide practical guidance for others in the field. Its ultimate purpose is to ensure that PD workshops are more closely aligned with participatory principles and avoid the pitfalls of tokenism.

With its five main categories and numerous factors, the framework not only offers concrete tools and guidance but also highlights the inherent complexity of PD workshops—their processes, components, and impact. Importantly, the framework is intended to be a living tool that will evolve through widespread use, critical reflection, and collective refinement. Such ongoing development would signal its success as a catalyst for participatory co-production and inclusive design, embodying the very principles it seeks to promote.

## Supplementary Information

Below is the link to the electronic supplementary material.

ESM 1 (DOCX 32.3 KB)

## Data Availability

No datasets were generated or analysed during the current study.

## Abbreviations

MS: Multiple sclerosis
PD: Participatory design
PPI: Patient and public involvement
