# Supplementary material for: Key Components of Participatory Design Workshops for Digital Health Solutions: Nominal Group Technique and Feasibility Study
Source: J Healthc Inform Res. 2025 May 14;9(3):359–79. doi: 10.1007/s41666-025-00199-4 (PMC12290142; doi:10.1007/s41666-025-00199-4)
Supplement: Supplementary file 1 — (DOCX 32.3 KB) [file 41666_2025_199_MOESM1_ESM.docx]

**Appendix 1: Checklist to consider the single factors of the framework in design and conduct of participatory design workshops aiming at developing digital health solutions**

| **Category** | **Factors** | **Do’s** | **Examples** |
| --- | --- | --- | --- |
| **Participatory process** | - Plurality of opinion - Plurality of expertise - Justification of the approach - Ownership - Joint rules - Joint terminology - Joint goals - Confidence in participants - Privacy protection - Participation barriers - Social fields of power - Impact of status and roles - Expectation management | Encourage Plurality of Opinion:   - Create an environment where diverse opinions are welcomed and valued. - Facilitate open discussions to ensure all voices are heard.   Leverage Plurality of Expertise:   - Involve participants with varied expertise to enrich the process. - Recognize and utilize the unique skills and knowledge of each participant.   Justify the Approach:   - Clearly explain the rationale behind the chosen participatory methods. - Ensure participants understand the benefits and goals of the approach.   Foster Ownership:   - Encourage participants to take ownership of the process and outcomes. - Involve participants in decision-making to enhance their commitment.   Establish Joint Rules:   - Collaboratively set ground rules to guide the workshop. - Ensure rules are agreed upon and respected by all participants.   Develop Joint Terminology:   - Create a shared language to avoid misunderstandings. - Ensure all participants are familiar with key terms and concepts.   Set Joint Goals:   - Define clear, shared objectives for the workshop. - Align individual goals with the collective aims of the group.   Build Confidence in Participants:   - Provide support and encouragement to boost participants' confidence. - Recognize and celebrate contributions to foster a positive atmosphere.   Protect Privacy:   - Ensure participants' privacy is respected and protected. - Handle sensitive information with care and confidentiality.   Address Participation Barriers:   - Identify and mitigate barriers to participation. - Provide support to ensure inclusivity.   Navigate Social Fields of Power:   - Be aware of power dynamics and strive for equitable participation. - Facilitate discussions to balance influence among participants.   Consider Impact of Status and Roles:   - Acknowledge the impact of participants' status and roles. - Create opportunities for all participants to contribute meaningfully.   Manage Expectations:   - Set realistic expectations for the workshop outcomes. - Communicate clearly about what participants can expect. | **Think-Pair-Share process:**  Initial thoughts and impulses are silently noted or drawn up by the participants. This is followed by an initial exchange in small groups of two to three people. Only then are the topics collected in the plenary group and, if necessary, sorted, bundled, and processed further.  This approach ensures that input from quiet or less extroverted people is included in the workshop. Quieter voices are more likely to be heard, and the risk of social group dynamics influencing individual participants too early is reduced.  This Think-Pair-Share method is also very useful for reflecting on the participatory process itself. This is essential to check the extent to which participants feel heard and taken seriously.  In order to manage expectations, it is particularly interesting to see whether the impressions and evaluations within the group are similar or different. Encourage discussion and concretization of perspectives by asking questions. Helpful questions might include - What exactly did we do? - Did we achieve our goal? If not, why not? - What can we take with us a "lesson learned" from the process?  **Establish Joint Rules:**  Common rules for the way we talk and work together in a participatory way in the workshop can be developed together. This increases their acceptance and can also, especially at the beginning of the collaboration, support the process of getting to know each other and building trust.  We use a variation that requires a large foam cube. You have the participants count out loud, using numbers from 1-6. Then you roll the dice and depending on the number rolled, those who counted that number can add something that is important to them. For example, a 3 is rolled. Someone in the group of 3s says, "It's important to me that I can finish talking. Or: I would like us to practice saying 'Yes, and...' instead of 'But...', etc. All the statements are collected so that everyone can read them. Then the group prioritizes the six most important statements as the applicable rules. This can be done simply by assigning points. Each participant receives three points, which he or she can distribute as he or she sees fit. |
| **Involved persons and their roles** | - Participant characteristics - Participant motivation - Literacy (health literacy, digital literacy) - Empowerment of participant | Participant Characteristics Recognize and value the diversity of participants' backgrounds, experiences, and perspectives.  - Tailor activities and discussions to accommodate different learning styles and preferences. - Ensure inclusivity by considering factors such as age, gender, cultural background, and abilities.   Participant Motivation   - Understand and address the individual motivations of participants to keep them engaged. - Provide clear incentives and benefits for participation. - Create a positive and supportive environment that fosters intrinsic motivation.   Literacy (Health Literacy, Digital Literacy)   - Assess the literacy levels of participants in advance and prepare appropriate resources and support. - Use clear and accessible language, avoiding jargon and complex terminology. - Offer training and educational materials to enhance participants' literacy skills.   Empowerment of Participant   - Encourage participants to take an active role in the process and decision-making. - Provide opportunities for participants to develop and showcase their skills and knowledge. - Create a safe space where participants feel confident to express their ideas and opinions. | Especially when larger groups are involved, *participation facilitators* can make sure that everyone can participate and that no one is overlooked or ignored. These participation facilitators have no other role than to take care of participants by encouraging their active participation, explaining processes, or asking the moderator or the group to change the pace if necessary.  Participation facilitators should be trained in advance and have a broad understanding of what good participation is, how it can be achieved, and what social or other dynamics may prevent participation from reaching its full potential. Being a facilitator requires an appropriate attitude. |
| **Workshop definition** | - Purpose - Goals - Participatory planning - Role definition and tasks - Specific needs - Inclusion/exclusion criteria - Validation of inclusion/exclusion criteria - Workshop structure - Selection of suitable methods - Selection and preparation of material - Specification of the setting - Agenda - Budgeting, first check - Budgeting, second check | Purpose   - Clearly define the purpose of the workshop to ensure all participants understand its significance. - Communicate the purpose effectively to align everyone's expectations and efforts.   Goals   - Set specific, measurable, achievable, relevant, and time-bound (SMART) goals for the workshop. - Ensure that the goals are aligned with the overall purpose and objectives of the PD workshop.   Participatory Planning   - Involve participants in the planning process to ensure their needs and perspectives are considered. - Encourage collaborative decision-making to foster a sense of ownership and commitment.   Role Definition and Tasks   - Clearly define the roles and responsibilities of each participant.   Specific Needs   - Identify and address the specific needs of participants to ensure inclusivity and accessibility.   Inclusion/Exclusion Criteria   - Establish clear criteria for participant inclusion and exclusion to ensure a relevant and diverse group. - Communicate these criteria transparently to avoid misunderstandings.   Validation of Inclusion/Exclusion Criteria   - Adjust the criteria as needed based on feedback and changing circumstances.   Workshop Structure   - Design a clear and logical structure for the workshop to facilitate smooth progression. - Include a mix of activities to keep participants engaged and energized.   Selection of Suitable Methods   - Choose methods that are appropriate for the workshop's goals and participants' needs, competencies and capabilities. - Be flexible and ready to adapt methods based on participants' feedback and dynamics.   Selection and Preparation of Material   - Carefully select and prepare materials that support the workshop's objectives and activities. - Prepare and organize materials in advance to ensure smooth execution.   Specification of the Setting   - Specify the setting of the workshop, including location, layout, and necessary equipment.   Agenda   - Create a detailed agenda that outlines the schedule and activities of the workshop. - Share the agenda with participants in advance to help them prepare.   Budgeting, First Check   - Conduct an initial budgeting check to estimate the costs and allocate resources accordingly. - Ensure that the budget aligns with the workshop's goals, setting and available funding.   Budgeting, Second Check   - Perform a second budgeting check closer to the workshop date to confirm and adjust expenses. - Monitor and manage the budget throughout the workshop to avoid overspending. | Develop a detailed schedule of the event that participants receive in advance and that is visible to everyone on site during the event.  Include regular breaks and time for informal interaction among participants. Depending on the target group, opportunities for rest and relaxation might be important.  Use visualizations to support statements. These are fun and usually easy to understand. They also activate other senses than just text. Visualizations also activate senses other than text and promote memory.  Drawing is a great strategy for bringing to the forefront participants' explicit and implicit knowledge. So, draw a big picture - a rich picture - to develop a common understanding of the issue at stake (see: [Rich picture \| Methods and tools for co-producing knowledge](https://naturalsciences.ch/co-producing-knowledge-explained/methods/method_factsheets/rich_picture)). Furthermore, goals, actions or even important challenges can be drawn together with the participants and support a joint understanding of complex issues (e.g. drawing a problem tree, a solution tree). |
| **Setting** | - Availability of appropriate material - Environmental factors | Availability of Appropriate Material   - Ensure that all necessary materials and resources are well-prepared, available and accessible to participants. - Provide high-quality, relevant materials that support the workshop's objectives. - Prepare backup materials in case of unexpected shortages or issues.   Environmental Factors   - Choose a comfortable and conducive environment for the workshop, considering factors like lighting, seating, and acoustics. - Ensure the venue is accessible to all participants, including those with disabilities. - Consider external factors such as weather, noise, and interruptions that could impact the workshop. | When people with disabilities are involved, it is always essential and best to work with them: check the rooms or venue for wheelchair access (even small thresholds can be insurmountable for some people!) and other accessibility criteria such as lighting, screen size, and acoustics (the latter is important for people with hearing disabilities). |
| **Privacy and ethics including regulations** | - Ethical considerations - Ethics approval - Regulation compliance | Ethical Considerations   - Prioritize the health, well-being, safety and rights of participants throughout the process. - Ensure transparency and honesty in all communications and actions. - Consider the potential impacts of the workshop on participants and the broader community. - Address any ethical dilemmas promptly and thoughtfully.   Ethics Approval   - Seek ethics approval from relevant institutional review boards or ethics committees before starting the workshop. - Provide detailed documentation of the workshop's objectives, methods, and potential risks for ethics assessment. - Ensure that all ethical guidelines and standards are met and maintained throughout the process.   Regulation Compliance   - Familiarize yourself with and adhere to all relevant regulations and legal requirements. - Ensure that data collection, storage, and usage comply with privacy and data protection laws. - Regularly review and update practices to remain compliant with any changes in regulations. | Not everyone can or wants to express criticism or problems out loud for others to hear. One or more flipcharts can be used to create a quiet corner out of the way where people can write down critical things. The facilitator can announce in advance that he or she will look at the flipcharts at certain times and address the issues raised. |
